# Transient osteoporosis of pregnancy in a case of postpartum bilateral femoral neck fracture

**Published:** 2018-10

**Authors:** Kaveh Gharanizadeh, Alireza Mirzaei, Solmaz Piri, Mozhdeh Zabihiyeganeh

**Affiliations:** 1 *Bone and Joint Reconstruction Research Center, Shafa Orthopedic Hospital, Iran University of Medical Sciences, Tehran, Iran.*; 2 *National Association of Iranian Gynecologists and Obstetrician, Tehran, Iran* *.*

**Dear Editor**

Transient osteoporosis of pregnancy (TOP) is a rare, yet under-reported condition that threatens pregnant women in the third trimester of a usually uneventful pregnancy. It is known to be the consequence of drastic loss of bone mass and elevated rates of bone turnover caused by fetal consumption of calcium and vitamin D from the maternal skeleton (1, 2). Meanwhile, the transient nature of osteoporosis cannot generally be determined at the time of the event, mainly due to the lack of bone mineral density (BMD) history.

With respect to our published article “Undesired effect of excessive betamethasone administration during pregnancy: A rare case” in volume 16, issue 3 of your journal (3), we were highly concerned about TOP based on the characteristics of the fracture. However, due to lack of adequate information of previous BMD, this concern could not be supported at that time and postpartum bilateral femoral neck fracture was the reported diagnosis instead. Therefore, we prescribed teriparatide in addition to calcium and vitamin D supplementation for the management of low BMD of the patient, which was discontinued after three months due to the high cost of the drug for her.

In order to rule out the main cause of bilateral femoral neck fracture in our patient, we followed the patient for 30 months after the surgery, when her ability to walk was completely returned to normal and she had no other complaint as well. We repeated the BMD of the patients to find out if she needs any osteoporosis medication. An 11% improvement was observed in the last lumbar spine BMD of the patient when compared with the earlier BMD performed at the time of bilateral femoral neck fracture diagnosis (BMD=0.906 g/cm2, T score=-1.3 versus BMD=0.816 g/cm2, T score=-2.1, respectively).

According to the provided evidence, especially improvement of follow-up BMD, we are convinced that this case could be truly considered as TOP, which was resolved after pregnancy and elimination of its concomitant risk factors (vitamin D deficiency, immobility, and excess steroid consumption). 

Although we prescribed teriparatide for the management of low BMD of the patient, our evaluations revealed that osteoporosis will spontaneously resolve in such cases. Hence, the clinical message of these findings could be to avoid unnecessary osteoporosis treatment in premenopausal pregnant women through the definitive diagnosis of TOP.
